# Effect of active and heat-killed *Clostridium butyricum* on *in vitro* gas production, ruminal fermentation parameters, and microbiota at varying media pH levels

**DOI:** 10.5713/ab.24.0913

**Published:** 2025-08-12

**Authors:** Xinlong Zhang, Zhiyue Zhang, Hongxu Zhu, Guanghui Hu, Hangshu Xin, Jincheng Liu, Xu Lin, Xiaolai Xie, Peixin Jiao

**Affiliations:** 1 College of Animal Science and Technology, Northeast Agricultural University, Harbin, China

**Keywords:** Bacterial Communities, *Clostridium butyricum*, Gas Production, Media pH Level, Ruminal Fermentation

## Abstract

**Objective:**

The objective of this study was to evaluate the effects of inactive (heat-killed) *Clostridium butyricum* (ICB) on gas production kinetics, fermentation parameters, and microbiota with varying media pH levels in batch culture.

**Methods:**

The *in vitro* experiment was designed as a completely randomized factorial arrangement, with 2 media pH levels (5.8 and 6.5)×2 *Clostridium butyricum* (CB) products (active and inactive)×4 dosages of CB. Two lactating dairy cows with ruminal fistulas, fed a diet comprising 40% forage and 60% concentrate, served as donors for rumen inoculum. Following 24 h of incubation, the gas production, dry matter disappearance (DMD), volatile fatty acid (VFA), ammonia nitrogen (NH_3_-N) and microbial profile were measured to determine the effect of treatment on fermentation.

**Results:**

The gas volume (GV), DMD, total VFA concentration, NH_3_-N concentration, acetate concentration and microbial alpha diversity were inhibited when the media pH decreased from 6.5 to 5.8. Increasing the supplemental doses of ICB linearly increased the GV, DMD (trend) and butyrate proportion at media pH 6.5. Moreover, the increasing supplemental dose of active *Clostridium butyricum* (ACB) linearly increased GV, butyrate proportion and NH_3_-N concentration (trend) regardless of media pH, and linearly increased DMD, total VFA concentration and A:P ratio at media pH 6.5. Supplementing ICB decreased the relative abundance of *Actinobacteria* and *Butyrivibrio* in the fermentation fluid.

**Conclusion:**

Increasing media pH promotes rumen fermentation and alter bacterial community. Although both ACB and ICB have the potential to stimulate rumen fermentation in a dose-dependent manner, their effects change depending on media pH levels. Furthermore, both ACB and ICB rarely altered the rumen bacterial community.

## INTRODUCTION

Probiotics, defined as “live microorganisms,” are extensively used as cost-effective alternatives to antibiotics in livestock, as they are generally understood to enhance animal immunity and growth performance by secreting nutrients such as amino acids, digestive enzymes, vitamins, and growth factors [1,2]. However, the safety profile of live probiotics continues to be a topic of ongoing debate. Key concerns include the risk of systemic infections resulting from translocation, particularly in immunocompromised individuals and pediatric populations, the potential acquisition of antibiotic resistance genes, and the disruption of gut colonization in neonates [3]. To mitigate these risks, there is increasing interest in the use of nonviable microorganisms or microbial cell extracts as alternatives to live probiotics, with a focus on heat-killed bacterial strains. Heat-treated probiotic cells, cell-free supernatants, and purified bioactive components have demonstrated beneficial effects, including immunomodulation, protection against enteropathogens, and the maintenance of intestinal barrier integrity [4]. Previous study has shown that yeast derivatives (yeast cell wall components) could stimulate ruminal fermentation of beef cattle [5]. Moreover, Vyas et al reported that the effects of active dried and heat-killed dried yeast on rumen pH or ruminal fermentation characteristics were not different in beef heifers [6]. Thus, it can be seen that the viability of some probiotics may not be required to elicit the beneficial effects in animals.

*Clostridium butyricum* (CB), a gram-positive anaerobic bacterium, exhibits high tolerance to low pH, elevated bile salts, and high temperatures. It produces butyric acid, forms spores, and inhabits the intestines of healthy animals and humans [7]. There is evidence that CB could enhance growth performance and health of livestock [8] and poultry [9] by secreting and activities of digestive enzymes in the intestine through the modulation of gut microbial composition or its production of butyric acid. Moreover, our previous studies showed that CB could stimulate ruminal fermentation by modulating ruminal microbiota composition, whereas it was affected by various factors such as diet composition, supplemental dose and rumen pH [7,10]. Recent studies have reported that postbiotics from probiotics can also have beneficial effects on the host through microbial cell components, metabolites and intermediate and end-products of microbial fermentation [11]. However, it is still unknown that whether heat-killed CB would have beneficial effect on ruminants through its cell wall, cellular components, vitamins, organic acids or other microbial metabolites. We hypothesized that supplementing inactive (heat-killed) *Clostridium butyricum* (ICB) could beneficially influence rumen fermentation by altering the microbial composition, with its effects potentially varying at different media pH levels. Therefore, the objective of this study was to evaluate the effects of active *Clostridium butyricum* (ACB) and ICB on gas production kinetics, fermentation parameters, and microbiota with varying media pH levels in batch culture.

## MATERIALS AND METHODS

### *Clostridiu*m *butyricum* resource and substrate

The product of CB with a concentration of 1.0×10^9^ CFU/g was provided by the Langood Biological Biotechnology. The ICB samples were obtained according to the method by Vyas et al [6]. To verify if the ICB samples were successfully obtained, the number of ICB colonies was detected using the plate counting method, and was found to be less than 2.2×10^2^ CFU/g, as reported by Wang et al [12]. The substrate used in this study was a typical diet for lactation dairy cows in China, of which consisted of 45% forage and 55% concentrate (dry matter [DM] basis; Table 1).

### Experimental design and inoculum

All experimental procedures involving animals were examined and sanctioned by the Institutional Animal Care and Use Committee of Northeast Agricultural University (Harbin, China). Animals were cared and managed according to the experimental license (NEAUEC20240291).

The *in vitro* experiment was designed as a completely randomized factorial arrangement, with 2 media pH levels (5.8 and 6.5)×2 CB products (active and inactive)×4 dosages. The four dosages included a shared control group (no supplementation) plus three product-specific levels: for ACB, these were 6×10^6^, 1.2×10^7^, and 2.4×10^7^ CFU/bottle; for ICB, they were 6, 12, and 24 mg/bottle. Two lactating dairy cows with ruminal fistulas, fed a diet comprising 40% forage and 60% concentrate, served as donors for rumen inoculum.

### Batch culture procedures

Rumen contents were collected from multiple sites within the rumen 2 h following the morning feeding. The samples were pooled and immediately filtered through four layers of cheesecloth. The pH of ruminal fluid was measured using a pH meter (PB-10; Sartorius). The recorded pH values were 6.61 and 6.37 for the first and second run, respectively. The filtered ruminal fluid was stored in a sealed, insulated container, promptly transferred to the laboratory, and maintained in a 39°C water bath. The buffer was prepared according to the formula described by Goering and Van Soest [13]. The achievement of the two target pH levels was accomplished by adjusting the sodium bicarbonate volume in the buffer solution [7]. Similarly, the freshly prepared buffer was also heated to 39°C in a water bath.

For the batch culture setup, 125 mL glass bottles fitted with rubber stoppers and aluminum lids were utilized. Filter bags (F57; AnkomTechnology) were initially soaked in acetone for 10 min, rinsed, dried, and weighed. Approximately 0.5 g of substrate, ground to pass through a 1-mm screen, was loaded into each filter bag. The substrate-containing bags were then placed into the bottles. The ACB and ICB samples were added to the bottles in line with the designated supplemental dose. Three additional bottles containing rumen fluid and buffer were served as blanks for gas volume (GV) correction at each pH level. The batch culture was repeated in an additional run after 7 days.

Each bottle was prepared by adding 45 mL of buffer and 15 mL of strained rumen fluid, with carbon dioxide introduced to ensure an anaerobic atmosphere. The bottles were sealed with rubber stoppers and aluminum caps, then immediately placed in a pre-heated shaking incubator (SPH-2102C; Shanghai Shiping Experimental Equipment) at 39°C. Gas pressure measurements were taken at 3, 6, 9, 12, and 24 h of incubation using a pressure transducer (HT-935; Hongcheng Technology) fitted with a 23-gauge needle (0.6 mm) inserted through the rubber stoppers. The pressure readings were corrected for DM of the substrate incubated, as well as for the blank samples, and the GV was subsequently calculated using the equation of Romero-Pérez and Beauchemin [14]:


(1) $$\text{GV}=4.7047\times (\text{ga}s\;\text{pressure})+0.0512\times (\text{gas }{\text{pressure}}^{2}).$$

The kinetic parameters of GV were estimated by fitting GV data to an exponential model [15]:


(2) $$\text{y}=\text{GV}\times (1-{\text{e}}^{-\text{c}\times [\text{t}-\text{lag}]})$$

Where “y” is the cumulative volume of gas produced at the time “t” (h), “GV” is the asymptotic GV, “c” is the constant fractional rate of GV, and “lag” is the time (h) between inoculation and commencement of GV.

Average gas production rate (AGVR, mL gas/h) from the onset of incubation to the point where cumulative GV reached half of its asymptotic value was calculated using the equation established by García-Martínez et al [16]:


(3) AGVR=(GV×c)/(2×[Ln2+c×Lag])

Following 24 h of incubation, the bottles were transferred to cold water to terminate fermentation. The pH of the fermentation fluid was measured with a portable pH meter (PB-10; Sartorius) after the removal of rubber stoppers and aluminum caps. Ten mL of fermentation fluid was collected from each bottle and divided into 2 parts, which were stored at −20°C with 1 mL of metaphosphoric acid (25%, w/v) and sulfuric acid (1%, v/v), respectively, for the later analysis of volatile fatty acid (VFA) and ammonia nitrogen (NH_3_-N) concentration. Additionally, separate samples from three bottles containing fermentation media with control and varying levels of ACB or ICB were collected, and 5 mL of fermentation fluid was extracted from each. These samples were immediately frozen in liquid nitrogen and stored at −80°C until DNA extraction.

### Microbial DNA isolation and 16S rRNA high throughput sequencing

Total genomic DNA was extracted from the fermentation fluid using the OMEGA Soil DNA Kit (M5635-02) (Omega Bio-Tek) following the manufacturer’s protocol. The DNA quantity and quality were evaluated using a NanoDrop NC2000 spectrophotometer and agarose gel electrophoresis, respectively. The PCR amplification was performed using the forward primer 338F (5’-ACTCCTACGGGAGGCAGCA-3’) and the reverse primer 806R (5’-GGACTACHVGGGTWTC TAAT-3’). Equal amounts of amplicons were pooled, and paired-end sequencing (2×250 bp) was performed on the Illumina NovaSeq platform using the NovaSeq 6000 SP Reagent Kit (500 cycles) at Shanghai Personal Biotechnology.

### Chemical analyses

The substrate were analyzed for DM (method 930.15), crude protein (CP, method 990.03), ether extract (EE, method 920.39) and ash (method 942.05) according to AOAC [17]. The contents of heat-stable α-amylase-treated neutral detergent fiber (NDF) and acid detergent fiber (ADF) in substrate were analyzed according to the method described by Van Soest et al [18].

The fermentation fluid VFA concentration was analyzed according to the method described by Liang et al [19]. Six mL of fermentation fluid fixed with metaphosphoric acid was centrifuged at 10,000×g for 15 min, and 1 mL of the supernatant was filtered through a 0.45 μm filter membrane and measured using a gas chromatograph (GC-2010; Shimadzu) equipped with 50 m capillary column. The temperatures of injector oven, column oven and detector were 230°C, 160°C and 240°C, respectively. NH_3_-N concentration of fermentation fluid was determined according to the method described by Broderick and Kang [20]. The 6 mL of fermentation fluid fixed with sulfuric acid was centrifuged at 12,000×g for 20 min, and 40 μL of the supernatant was mixed with 2.5 mL phenol chromogenic agent and 2 mL hypochlorite solution in turn, and the color was developed in a water bath at 37°C for 30 min.

### Statistical analysis

Model validation was performed using diagnostic plots, including fitted residuals and Q-Q plots, with R software. Where it was required, Box-Cox transformation was applied to enhance homogeneity and normality, and models were re-fitted using the transformed data. All data were analyzed using PROC MIXED procedure of SAS (SAS Institute) with a model that included fixed effects of media pH, CB, supplemental dose of CB, two-way and three-way interactions, and the random effects of run. Tukey’s multiple comparison test was used to examine the significance of ACB and ICB among different supplemental doses (control, low, medium and high). Moreover, contrasts were generated to allow comparisons among the average of control, ACB and ICB. The CONTRAST statement of SAS with linear orthogonal contrasts was used to determine the effect of increasing the dose of CB. Differences were declared significant at p≤0.05, and trends were discussed at 0.05<p≤0.10.

## RESULTS

### Gas production kinetics and dry matter disappearance

Three-way interaction among pH, dose and CB was noticed for GV (p = 0.03). Moreover, pH was interacted (p<0.01) with dose for all the gas kinetic parameters except for lag time. The gas kinetic parameters of GV, rate constant, lag time and AGVR were consistently greater (p<0.01) at media pH 6.5 than pH 5.8 (Table 2). In comparison with ICB, the supplementation of ACB increased GV (p = 0.02) and AGVR (p = 0.02) at media pH 5.8. Similarly, the ACB supplementation increased GV (trend, p = 0.08) and AGVR (p = 0.02), while decreasing lag time compared to ICB at media pH 6.5. The GV, lag time and AGVR linearly (p<0.01) increased with increasing supplemental dose of ACB at both media pH levels; whereas, they linearly (p<0.01) increased with increasing supplemental dose of ICB at media pH 6.5.

An interaction (p = 0.02) between pH and supplemental dose of CB was noticed on DMD. The increase of media pH from 5.8 to 6.5 increased (p<0.01) DMD. At media pH 6.5, the DMD was greater (p = 0.01) with supplementation of ACB compared to ICB; whereas, it was not different between supplementation of ACB and ICB at media pH 5.8. The DMD was linearly increased with increasing supplemental dose of ACB (p<0.01) or ICB (trend, p = 0.06) at media pH 6.5.

### Ruminal fermentation characteristics

There was no interaction among pH, supplemental does and CB on total VFA concentration, proportions of acetate, propionate, and BCVFA, or the acetate: propionate (A:P) ratio (Table 3). Nevertheless, CB interacted with pH levels, dose, or a combination of both for butyrate. Moreover, an interaction (p = 0.03) between pH and dose was observed for NH_3_-N. The TVFA concentration (p<0.01), acetate proportion (p = 0.03), and NH_3_-N concentration (p<0.01) were higher, while the butyrate proportion was lower (p<0.01) at media pH 6.5 compared to pH 5.8. The higher (p<0.01) butyrate proportion and lower (trend, p = 0.06) propionate proportion were observed with the supplementation of ACB compared to ICB at media pH 5.8. Additionally, the higher (p<0.01) NH_3_-N concentration and lower (trend, p = 0.08) propionate proportion were observed at media pH 6.5. At media pH 5.8, the butyrate proportion increased (linear; p<0.01) and the acetate proportion decreased (linear; p = 0.03) with increasing doses of ACB, whereas increasing the supplemental dose of ICB had no effect on fermentation characteristics. At media pH 6.5, the total VFA concentration (linear; p<0.01) and A:P ratio (linear; p<0.01) increased, while the propionate proportion (linear; p<0.01) decreased with increasing dose of ACB. Moreover, increasing the supplemental dose of ICB quadratically changed (p<0.01) the butyrate proportion at media pH 6.5.

### Microbial diversity analysis

**Figure 1:**
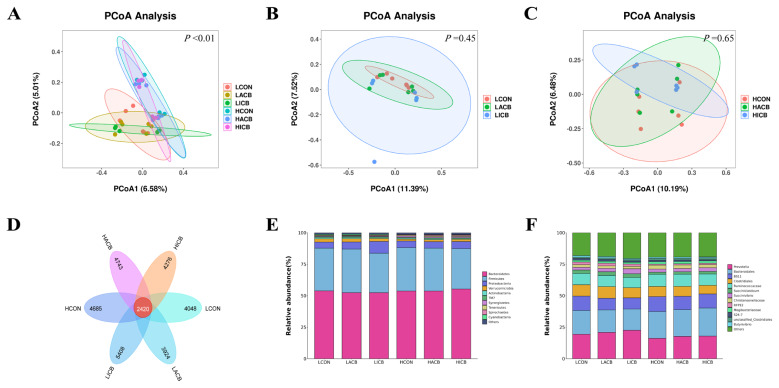
Effects of media pH and CB products on ruminal bacterial communities. (A) Principal coordinates analysis (PCoA) of rumen fluid at different pH levels; (B) PCoA of rumen fluid of different CB products at pH 5.8; (C) PCoA of rumen fluid of different CB products at pH 6.5; (D) Analysis of ASVs’ exclusiveness of microbiome of ruminal fermentation fluid; (E) Relative abundance of ruminal microbiota at the phylum-level; (F) Relative abundance of ruminal microbiota at the genera-level. LCON, supplemented without ACB or ICB at low media pH (5.8); LACB, supplemented with 2.4×10^7^ CFU/bottle of ACB at low media pH (5.8); LICB, supplemented with 24 mg/bottle of ICB at low media pH (5.8); HCON, supplemented without ACB or ICB at high media pH (6.5); HACB, supplemented with 2.4×10^7^ CFU/bottle of ACB at high media pH (6.5); HICB, supplemented with 24 mg/bottle of ICB at high media pH (6.5). CB, *Clostridium butyricum*; ACB, active *Clostridium butyricum*; ICB, inactive (heat-killed) *Clostridium butyricum*.

There was no interaction between pH and CB for the alpha diversity index (Table 4). The indexes of Pielou_evenness (p = 0.04), Shannon (p = 0.03) and Simpson (trend, p = 0.06) were higher at media pH 6.5 than pH 5.8. Furthermore, ACB supplementation increased Shannon index compared to ICB supplementation at media pH 6.5 (p = 0.02). In order to analyze the Beta diversity of the bacterial communities in the fermentation fluid, principal coordinates analysis (PCoA) was conducted and illustrated based on jaccard distance matrices to characterize the clustering of bacterial microbiota between two pH levels without adding CB, or between control, ACB and ICB at media pH 5.8 or pH 6.5 (Figures 1A–1C), respectively. PCoA revealed a clear separation between the two groups of bacterial communities at media pH 5.8 and media pH 6.5. Moreover, at the media pH 5.8 and media pH 6.5, the rumen bacterial aggregation of different CB was better, and there was no significant difference. The Venn diagram in the fermentation fluid (Figure 1E) showed that LCON, LACB, LICB, HCON, HACB and HICB shared 2,420 ASVs, and had 4,048, 3,924, 5,408, 4,685, 4,743 and 4,276 exclusive ASVs.

### Bacterial composition in the fermentation

The top 10 relative abundance of microbiota in the fermentation fluid was determined at the phylum level (Table 5, Figure 1F). No interaction between pH and CB was observed in the abundances of the microbiota. In comparison with media pH 5.8, the abundances of *Verrucomicrobia* (p<0.01), *Actinobacteria* (p = 0.01), *TM7* (p = 0.01) and *Cyanobacteria* (p<0.01) were lower, whereas the abundances of *Synergistetes* (p<0.01), *Tenericutes* (p<0.01) and *Spirochaetes* (p<0.01) were higher at media pH 6.5. Supplementation of ICB increased (p = 0.03) the *Spirochaetes* abundance and decreased (p<0.01) the *Actinobacteria* abundance at media pH 5.8. In contrast, ICB supplementation increased the *Firmicutes* abundance (trend, p = 0.07) and *Firmicutes:Bacteroidetes* (F:B) ratio (trend, p = 0.09) at media pH 6.5 compared to ACB supplementation.

At the genus level, the top 13 microbiota based on relative abundance (>1% in at least one raw sample from a group) were detected in the fermentation fluid (Table 6). There was no interaction between pH and CB in the abundances of the microbiota. The abundances of *Bacteroidales* (p<0.01), *BS11* (p = 0.04), *Christensenellaceae* (p<0.01), *[Mogibacteriaceae]* (p = 0.03), and *unclassified_Clostridiales* (p<0.01) were higher, whereas the abundances of *Prevotella* (p = 0.02), *Succiniclasticum* (p<0.01), and *RFP12* (p<0.01) was lower at media pH 6.5 than pH 5.8. The ICB addition tended (p = 0.07) to decrease the abundance of *Butyrivibrio* at media pH 5.8, and decreased the abundances of *Christensenellaceae* (trend, p = 0.06), *S24-7* (trend, p = 0.07) and *Butyrivibrio* (p = 0.01) at media pH 6.5 compared to ACB.

## DISCUSSION

### Gas production, dry matter disappearance, and fermentation characteristics

Rumen pH plays a crucial role in affecting the microbial population and fermentation end products [21]. It is reported that the rumen pH 6.0 would be considered a threshold value for subacute rumen acidosis. The mean pH ranged from 5.89 to 6.63 in the rumen when the dairy cows were fed a corn silage based total mixed ration [22]. Therefore, based on our previous studies, we selected target pH levels of 5.8 (low) and 6.5 (high) to evaluate differential ACB and ICB effects across ruminal pH conditions [5,23]. In the current study, the final media pH was 5.91 and 6.67 after 24 h of incubation, which was close to our target low (5.8) and high (6.5) media pH. This suggests a high buffering capacity of the fermentation media, despite the acids produced during fermentation. The GV, DMD, and total VFA consistently increased at pH 6.5 versus 5.8, indicating enhanced feed degradation under higher ruminal pH. Our previous studies showed that low media pH would inhibit the growth of *fibrolytic* bacteria such as *Fibrobacter succinogenes* and *Ruminococcus flavefaciens*, which may be responsible for the decrease of GV, DMD and total VFA concentration at media pH 5.8 versus pH 6.5 in the present study [5]. In consistent with current study, our previous studies revealed that the GV, DMD and total VFA were overall greater at high media pH than low media pH in batch culture [5,7].

In the present study, the GV and DMD linearly increased with increasing supplemental dose of ACB at media pH 6.5, the result is consistent with Zhang et al [10], who reported a linear increase in gas production (24 h of incubation) and DMD with increasing supplemental dose of CB on *in vitro* using rumen inoculum from dairy cows. It is reported that ACB is an endospore-forming bacteria, which could produce short-chain fatty acids and nutritional factors such as enzymes and vitamins, which would be favorable for increasing the activities of *fibrolytic* bacteria in the rumen [24]. Moreover, the total VFA concentration linearly increased with increasing dose of ACB at media pH 6.5 in the present study, and the result is consistent with DMD. The greater butyrate proportion with ACB supplementation at both media pH levels could be a result of butyrate produced by ACB. NH_3_-N can be used by rumen microorganisms to synthesize microbial protein [24]. Our previous study showed that CB increased the concentration of NH_3_-N by increasing DMD [10]. We obtained consistent results at pH 6.5; however, the NH_3_-N concentration tended to decrease with increasing supplemental dose of ACB, suggesting the pH would be an important factor affecting the impact of CB on NH_3_-N concentration. In addition, a lower NH_3_-N may indicate that the utilization of NH_3_-N by microorganisms is improved when the DMD remains unchanged.

In batch cultures using rumen inoculum, the fermentation of feed substrates results in the production of VFA, gas, and microbial biomass. The VFA and microbial biomass generated during rumen fermentation serve as valuable energy and protein sources for ruminants, while gas represents an energy loss, as it is not utilized by the animal. Thus, the linearly increased GV and DMD (trend) with no difference in total VFA concentration at media pH 6.5 was unexpected. Up to now, little work has been done to investigate the effects of ICB on rumen fermentation characteristics. Studies have indicated that the structural components of probiotic cells, especially membrane-associated structures such as the cell wall, capsule, pellicle, and Slayer proteins, are essential in modulating the host’s immune response [3]. These cellular structures are key in mediating interactions between probiotics and the host, influencing immune activation and overall immune system function [25]. Furthermore, Adams suggested that the primary means of the modulation by components of dead cells included nonviable material that originates from microorganisms [26]. Thus, we speculated that supplementation of ICB may change the rumen fermentation characteristics by its membrane components. However, further study is warranted to understand the mechanisms of which and how membrane component affects rumen fermentation. In addition, the DMD was not different between ACB and ICB at media pH 5.8, whereas it was greater with ACB compared to ICB at media pH 6.5. Zhang et al found the DMD was greater with ACB supplementation at media pH 6.6 on *in vitro* rumen fermentation but not at media pH 6.0, suggesting that high media pH would favor the activity of ACB in the rumen [10]. Thus, this pH-dependent efficacy difference likely stems from enhanced ACB activity at pH 6.5.

### Ruminal bacterial microbiota

The rumen microbial diversity, dominance and richness are regarded as the key elements that can reflect the function of the rumen [27]. At pH 5.8, reduced alpha diversity indexes (Chao 1, Shannon, Simpson, ASVs) indicated impaired rumen function. This aligns with lower GV, DMD, and total VFA versus pH 6.5. The effects of ACB supplementation on alpha diversity indexes have been inconsistent in previous studies. Zhang et al reported that supplementation of ACB did not change the alpha diversity indexes on *in vitro* rumen fermentation using a substrate consisted of 45% concentrate and 55% forage [10]. Similarly, the alpha diversity indexes of Chao 1, Shannon, Simpson and ASV number were not influenced by the supplementation of ACB or ICB in the current *in vitro* study, which used a substrate consisted of 45% concentrate and 55% forage. In contrast, when a high-concentrate diet (60% concentrate and 40% forage) supplemented with ACB was fermented *in vitro*, the alpha diversity indexes (OTU, Shannon, Simpson, Chao1, and Evenness) were generally higher at the elevated media pH compared to the lower media pH [7]. Therefore, it can be speculated that the effects of ACB on alpha diversity indexes in the rumen may be associated with the dietary formulation, particularly the concentrate-to-forage ratio. In this study, the PCoA analysis revealed that the bacterial communities were clearly separated due to significant alterations in rumen microbial composition at two media pH levels, which was consistent with our previous study [10]. However, the clustering of bacterial microbiota overlapped between the control, ACB, and ICB groups at both media pH 5.8 and pH 6.5, indicating that CB has a limited effect on microbiota diversity, regardless of its activity.

*Firmicutes* and *Bacteroidetes* are the dominant phyla in ruminal microbial community regardless of media pH levels or CB supplementation, which is consistent with the previous results presented in *in vivo* or *in vitro* studies [7,8]. Li et al reported that rumen pH is the most known abiotic factor that influences rumen bacteria community, particularly through inhibition pH-sensitive cellulolytic bacteria [28]. Most of the abundances of identified bacteria were altered either in phylum (7/10) or genus level (11/14) in the current study. Similarly, Guo et al. found that the abundances of the most identified bacteria either at phylum (e.g. *Firmicutes*, *Proteobacteria*, *Spirochaetes* and *Actinobacteria*) or genus (e.g. *Prevotella*, *Pseudobutyrivibrio*, *Olivibacter* and *Lactobacillus*) level were changed at media pH 6.5 group compared to media pH 5.8 group in a Rusitec system [29].

It is reported that dietary ACB can modulate the gastrointestinal bacterial communities of livestock [8] and poultry [30] through its probiotic properties. However, there are few studies on the effects of ICB on the bacterial communities of animals, it remains unclear whether CB activity is necessary to exert an influence on the microbial community. In the current study, bacterial communities showed little difference between ACB and ICB supplementation at both the phylum and genus levels, regardless of media pH, suggesting that CB, with or without activity, has a similar effect on ruminal bacterial communities. *Actinobacteria* are a group of gram-positive bacteria which colonize and also actively influence the digestive system of animals, including ruminants [31]. Although role of *Actinobacteria* in rumen fermentation is still limited, Guo et al. found that *Actinobacteria* could survive and increase under a subacute ruminal acidosis condition and low media pH stimulated their growth and activity, which is consistent with the current study [29]. In the rumen ecosystem, *Spirochaetes* are involved in the breakdown of complex substrates like cellulose, pectin, and phytic acid. They also participate in the fermentation of fermentable carbohydrates and contribute to the production of VFAs, including propionate, which are essential for the energy metabolism of ruminants [32,33]. Therefore, the greater propionate proportion with supplementation of ICB could be partly explained by the increased abundance of *Spirochaetes* compared to ACB supplementation in the current study. *Butyrivibrio* is a strictly anaerobic gram-positive bacterium [34]. Studies have shown that ACB can promote the proliferation of anaerobic bacteria by producing carbon dioxide to enhance the anaerobic environment in the fermentation bottle [35]. This may explain why the relative abundance of *Butyrivibrio* in ACB was higher than that in ICB in the current study. Previous study has demonstrated that *Butyrivibrio* play a critical role in the carbon flow within the rumen by initiating the breakdown of lignocellulose. They further metabolize the resulting by-products into short-chain fatty acids and other fermentation end products, which are essential for the energy metabolism of ruminants [36]. The decreased abundance of *Butyrivibrio* with supplementation of ICB may be detrimental to fiber degradation in the current study.

## CONCLUSION

The GV, DMD, and total VFA concentrations were consistently higher at media pH 6.5 than at pH 5.8, likely due to the inhibition of pH-sensitive cellulolytic bacteria at low media pH. Both ACB or ICB have the potential to stimulate rumen fermentation by increasing GV, DMD or total VFA concentration in a dose-dependent manner at higher media pH (6.5). In comparison with ACB, supplementation of ICB decreased GV, DMD (pH 6.5), NH_3_-N concentration, and butyrate proportion (pH 5.8), while increasing propionate proportion, indicating distinct effects on rumen fermentation. However, the bacterial community was rarely altered, suggesting that the activity of CB may be important for stimulating rumen fermentation, but not necessarily required to modulate rumen microbiota at the doses used in this study.

## Figures and Tables

**Table 1:** Ingredients and chemical composition of experimental substrate

Item	Content
Ingredients (%)	
Alfalfa hay	9.05
Oat hay	3.35
Corn silage	33.62
Corn grain	20.10
Soybean meal	9.64
Steam-flaked corn	6.02
Molasses	5.48
Cotton seeds meal	3.92
Expanded soybean	3.52
Canola seed meal	1.57
Fat powder	1.25
Cottonseed meal	1.17
Sodium bicarbonate	0.70
Premix^1)^	0.61
Chemical composition (% of DM)	
Dry matter	88.24
Organic matter	92.12
Crude protein	16.55
Ether extract	8.88
Neutral detergent fiber	47.38
Acid detergent fiber	24.85
Calcium	0.81
Phosphorus	0.38
Metabolic energy^2)^ (MJ/kg)	8.91

1) Supplied per kilogram of dietary DM: vitamin A, 16,000 IU; vitamin D3, 1,400 IU; vitamin E, 100 IU; Cu, 8.5 mg; Fe, 50 mg; Zn, 45 mg; Mn, 27 mg; I, 0.5 mg; Se, 0.5 mg; Co, 0.4 mg.

2) Metabolic energy was a calculated value.

**Table 2:** Effects of media pH and supplemental doses and types of CB on GV kinetics and DMD

Items	CON^1)^	ACB^2)^			ICB^3)^			SEM	p-value^4)^							

									pH	CB	Linear		pH× CB	pH× Dose	CB× Dose	pH× CB× Dose
		
		Low	Med	High	Low	Med	High				Active	Inactive				
pH 5.8																
GV (mL/g DM)	170	170	173	180	169	162	168	11.1	<0.01	0.02	<0.01	0.50	0.73	<0.01	0.22	0.03
C (%/h)	8.93	8.64	9.41	10.08	9.22	8.94	9.71	0.687	<0.01	0.78	<0.01	0.23	0.64	<0.01	0.96	0.51
Lag (h)	1.66	1.36	1.52	1.61	1.76	1.60	1.94	0.218	<0.01	0.14	0.90	0.32	0.82	0.33	0.56	0.99
AGVR (mL/h)	9.08	9.26	9.83	10.77	9.37	8.83	9.30	1.156	<0.01	0.02	<0.01	0.87	0.56	<0.01	0.47	0.17
DMD (%)	56.7	53.9	56.0	55.2	55.4	54.8	55.7	0.86	<0.01	0.66	0.15	0.46	0.02	<0.01	0.74	0.06
pH 6.5																
GV (mL/g DM)	209^c^	246^bc^	262^ab^	267^ab^	231^c^	253^ab^	272^a^	8.7	<0.01	0.08	<0.01	<0.01	0.73	<0.01	0.22	0.03
C (%/h)	12.4^d^	15.3^a^	16.3^a^	16.8^a^	14.6^b^	16.1^a^	16.7^a^	0.69	<0.01	0.39	<0.01	<0.01	0.64	<0.01	0.96	0.51
Lag (h)	2.02	2.19	2.31	2.13	2.49	2.39	2.39	0.218	<0.01	0.04	0.61	0.08	0.82	0.33	0.56	0.99
AGVR (mL/h)	13.7^e^	18.1^c^	19.8^abc^	21.3^a^	15.9^d^	18.9^bc^	20.8^ab^	0.74	<0.01	0.02	<0.01	<0.01	0.56	<0.01	0.47	0.17
DMD (%)	60.7^bc^	62.7^ab^	63.7^a^	64.4^a^	58.7^c^	63.0^ab^	62.1^abc^	1.19	<0.01	0.01	<0.01	0.06	0.02	<0.01	0.74	0.06

1) CON, control was supplemented without ACB or ICB.

2) ACB was supplemented with ACB; Low, med, and high of ACB group were supplemented, respectively, with 0.6×10^7^, 1.2×10^7^, and 2.4×10^7^ CFU/bottle of ACB.

3) ICB was supplemented with ICB; Low, med, and high of ICB group were supplemented, respectively, with 6, 12, 24 mg/bottle of ICB.

4) pH = pH 5.8 vs. 6.5, comparison among media pH 5.8 and 6.5; CB = ACB vs. ICB, comparison among ACB and ICB; Linear (Active), linear effect of ACB supplementation; Linear (Inactive), linear effect of ICB supplementation; pH×CB, interaction between media pH and CB products; pH×Dose, interaction between media pH and supplemental dose; CB×Dose, interaction between CB products and supplemental dose; pH×CB×Dose, interaction between media pH, CB products and supplemental dose.

a–e Means within a row with different superscripts differ (p<0.05).

CB, *Clostridium butyricum*; GV, asymptotic gas volume; DMD, dry matter disappearance; CON, control; ACB, active *Clostridium butyricum*; ICB, inactive (heat-killed) *Clostridium butyricum;* SEM, standard error of the mean; C, rate constant of GV; Lag, initial delay before GV begins (h); AGVR, average gas production rate.

**Table 3:** Effects of media pH and supplemental doses and types of CB on fermentation characteristics

Items	CON^1)^	ACB^2)^			ICB^3)^			SEM	p-value^4)^							

									pH	CB	Linear		pH× CB	pH× Dose	CB× Dose	pH× CB× Dose
		
		Low	Med	High	Low	Med	High				Active	Inactive				
pH 5.8																
TVFA (mM)	87.7	87.1	89.3	92.6	86.4	87.8	93.2	3.93	<0.01	0.82	0.26	0.20	0.83	0.34	0.78	0.56
VFA profiles (mol/100 mol)																
Acetate	53.3	52.5	52.3	51.5	52.0	52.1	53.2	0.71	0.03	0.54	0.03	0.79	0.17	0.54	0.98	0.06
Propionate	27.6	27.4	27.2	27.0	27.9	27.7	27.5	0.42	0.41	0.06	0.23	0.65	0.97	0.67	0.40	0.61
Butyrate	12.4^d^	13.5^bc^	14.0^b^	15.2^a^	13.3^bc^	13.5^bc^	13.0^cd^	0.314	<0.01	<0.01	<0.01	0.24	<0.01	0.07	0.01	0.01
BCVFA	3.39	3.35	3.26	3.13	3.39	3.35	3.15	0.106	0.06	0.50	0.05	0.07	0.58	0.23	0.21	0.15
A:P (ratio)	1.94	1.92	1.92	1.91	1.87	1.89	1.94	0.050	0.09	0.51	0.72	0.67	0.44	0.63	0.70	0.19
NH_3_-N (mM)	18.14	18.32	17.71	17.77	17.81	18.08	17.50	0.243	<0.01	0.49	0.06	0.07	0.14	0.03	0.11	0.76
pH 6.5																
TVFA (mM)	92.4	96.1	99.7	107.8	100.4	103.2	101.2	4.28	<0.01	0.88	<0.01	0.15	0.83	0.34	0.78	0.56
VFA profiles (mol/100 mol)																
Acetate	53.4	52.8	53.7	54.2	53.0	53.3	52.0	0.78	0.03	0.16	0.18	0.16	0.17	0.54	0.98	0.06
Propionate	27.7	27.5	27.1	26.3	27.7	27.2	27.5	0.38	0.41	0.08	<0.01	0.57	0.97	0.67	0.40	0.61
Butyrate	12.1	12.6	12.4	13.1	12.4	12.8	13.0	0.26	<0.01	0.82	0.01	<0.01	<0.01	0.07	0.01	0.01
BCVFA	3.42	3.53	3.38	3.20	3.41	3.30	3.87	0.202	0.06	0.34	0.06	0.13	0.58	0.23	0.21	0.15
A:P (ratio)	1.93	1.92	1.99	2.06	1.92	1.96	1.89	0.053	0.09	0.10	<0.01	0.62	0.44	0.63	0.70	0.19
NH_3_-N, mM	19.06	19.19	19.59	19.52	18.36	19.24	18.89	0.332	<0.01	<0.01	0.05	0.77	0.14	0.03	0.11	0.76

1) CON, control was supplemented without ACB or ICB.

2) ACB was supplemented with ACB; Low, med, and high of ACB group were supplemented, respectively, with 0.6×10^7^, 1.2×10^7^, and 2.4×10^7^ CFU/bottle of ACB.

3) ICB was supplemented with ICB; Low, med, and high of ICB group were supplemented, respectively, with 6, 12, 24 mg/bottle of ICB.

4) pH = pH 5.8 vs. 6.5, comparison among media pH 5.8 and 6.5; CB = ACB vs. ICB, comparison among ACB and ICB; Linear (Active), linear effect of ACB supplementation; Linear (Inactive), linear effect of ICB supplementation; pH×CB, interaction between media pH and CB products; pH×Dose, interaction between media pH and supplemental dose; CB×Dose, interaction between CB products and supplemental dose; pH×CB×Dose, interaction between media pH, CB products and supplemental dose.

a–d Means within a row with different superscripts differ (p<0.05).

CB, *Clostridium butyricum*; CON, control; ACB, active *Clostridium butyricum*; ICB, inactive (heat-killed) *Clostridium butyricum*; SEM, standard error of the mean; TVFA, total volatile fatty acids; BCVFA, branch-chained VFA (isobutyrate+isovalerate); A:P ratio, acetate:propionate ratio.

**Table 4:** Effects of media pH and different types of CB on alpha diversity of ruminal bacterial communities of rumen fermentation broth *in vitro*

Items	pH 5.8^1)^			pH 6.5			SEM	p-value^2)^			

								pH	CB		pH×CB
	
	CON	ACB	ICB	CON	ACB	ICB			Low	High	
ASVs	3,514	3,345	3,619	3,618	3,721	3,512	158.1	0.34	0.31	0.20	0.32
Goods_coverage	0.98	0.98	0.98	0.98	0.98	0.98	0.002	0.96	0.33	0.36	0.38
Chao1	4,046	3,826	4,214	4,106	4,303	4,045	206.9	0.47	0.27	0.23	0.30
Faith-pd	181.6	190.4	193.4	190.0	198.4	195.6	12.88	0.56	0.89	0.88	0.96
Pielou_evenness	0.86	0.85	0.84	0.86	0.86	0.86	0.007	0.04	0.35	0.30	0.49
Shannon	10.10	9.96	9.89	10.19	10.21	10.09	0.103	0.03	0.48	0.02	0.66
Simpson	0.998	0.997	0.996	0.998	0.998	0.998	0.0005	0.06	0.17	0.25	0.17

1) CON, control was supplemented without ACB or ICB; ACB was supplemented with 2.4×10^7^ CFU/bottle of ACB; ICB was supplemented with 24 mg/bottle of ICB.

2) pH = pH 5.8 vs. pH 6.5, comparison among treatments of control and CB products at media pH 5.8 and pH 6.5; CB = ACB vs. ICB, comparison among ACB and ICB products at low (pH 5.8) or high media (pH 6.5); Low, comparison among ACB and ICB products at low media pH (5.8); High, comparison among ACB and ICB products at high media pH (6.5); pH×CB, the interaction between media pH and CB products.

CB, *Clostridium butyricum*; CON, control; ACB, active *Clostridium butyricum*; ICB, inactive (heat-killed) *Clostridium butyricum*; SEM, standard error of the mean.

**Table 5:** The relative abundance (%) of bacterial phylum (top 10) in medium supplemented with CB and without CB (control) at low or high media pH after fermentation

Phylum	pH 5.8^1)^			pH 6.5			SEM	p-value^2)^			

								pH	CB		pH×CB
		
	CON	ACB	ICB	CON	ACB	ICB			Low	High	
*Bacteroidetes*	53.88	52.47	52.44	53.74	53.73	55.35	1.181	0.11	0.99	0.07	0.31
*Firmicutes*	33.88	34.52	31.32	34.48	34.01	32.05	2.180	0.77	0.19	0.14	0.94
*Proteobacteria*	4.81	5.68	9.39	5.25	5.19	5.63	1.625	0.53	0.27	0.64	0.60
*Verrucomicrobia*	2.62^a^	2.61^a^	2.25^ab^	1.44^c^	1.63^c^	1.72^bc^	0.203	<0.01	0.54	0.42	0.25
*Actinobacteria*	1.02^a^	1.01^a^	0.80^bc^	0.88^ab^	0.82^abc^	0.67^c^	0.097	0.01	<0.01	0.21	0.91
*TM7*	0.76^ab^	0.83^a^	0.67^bc^	0.61^c^	0.64^bc^	0.68^bc^	0.719	0.01	0.07	0.63	0.07
*Synergistetes*	0.52^b^	0.47^b^	0.47^b^	0.92^a^	0.88^a^	0.91^a^	0.049	<0.01	0.92	0.71	0.87
*Tenericutes*	0.50^b^	0.39^b^	0.46^b^	0.68^a^	0.73^a^	0.70^a^	0.066	<0.01	0.59	0.51	0.44
*Spirochaetes*	0.30^bc^	0.28^c^	0.53^ab^	0.44^abc^	0.69^a^	0.62^a^	0.089	<0.01	0.03	0.73	0.20
*Cyanobacteria*	0.59^a^	0.70^a^	0.56^a^	0.13^b^	0.17^b^	0.20^b^	0.057	<0.01	0.34	0.24	0.37
*Others*	1.14^b^	1.05^b^	1.11^b^	1.45^a^	1.53^a^	1.48^a^	0.094	<0.01	0.69	0.69	0.49
*F:B* ratio	0.64	0.68	0.60	0.64	0.64	0.58	0.052	0.80	0.24	0.09	0.90

1) CON, control was supplemented without ACB or ICB; ACB was supplemented with 2.4×10^7^ CFU/bottle of ACB; ICB was supplemented with 24 mg/bottle of ICB.

2) pH = pH 5.8 vs. pH 6.5, comparison among treatments of control and CB products at media pH 5.8 and pH 6.5; CB = ACB vs. ICB, comparison among ACB and ICB products at low (pH 5.8) or high media (pH 6.5); Low, comparison among ACB and ICB products at low media pH (5.8); High, comparison among ACB and ICB products at high media pH (6.5); pH×CB, the interaction between media pH and CB products.

a–c Means within a row with different superscripts differ (p<0.05).

CB, *Clostridium butyricum*; CON, control; ACB, active *Clostridium butyricum*; ICB, inactive (heat-killed) *Clostridium butyricum*; SEM, standard error of the mean; F:B ratio, *Firmicutes:Bacteroidetes* ratio.

**Table 6:** The relative abundance (%) of bacterial genus (top 13) in medium supplemented with CB and without CB (control) at low or high media pH after fermentation

Genus	pH 5.8^1)^			pH 6.5			SEM	p-value^2)^			

								pH	CB		pH×CB
	
	CON	ACB	ICB	CON	ACB	ICB			Low	High	
*Prevotella*	19.27	20.74	22.49	16.09	17.63	17.84	1.793	0.02	0.31	0.89	0.92
*unidentified_Bacteroidales*	18.68^bc^	17.65^c^	16.69^c^	21.15^ab^	21.10^ab^	22.17^a^	0.901	<0.01	0.53	0.32	0.25
*unidentified_BS11*	11.31	9.27	8.82	11.72	10.54	11.08	0.781	0.04	0.64	0.45	0.47
*unidentified_Clostridiales*	8.92	9.08	7.95	8.02	7.80	6.82	0.936	0.14	0.21	0.10	0.99
*unidentified_Ruminococcaceae*	8.91	8.89	8.11	9.51	9.55	8.90	0.755	0.12	0.19	0.26	0.94
*Succiniclasticum*	2.73^a^	2.82^a^	2.93^a^	1.75^b^	1.99^b^	2.05^b^	0.166	<0.01	0.65	0.63	0.77
*Succinivibrio*	2.11	2.65	3.92	2.43	2.52	2.92	0.858	0.91	0.62	0.64	0.98
*unidentified_Christensenellaceae*	2.11^bc^	2.04^c^	1.93^c^	2.99^a^	2.74^ab^	2.39^abc^	0.248	<0.01	0.52	0.06	0.85
*unidentified_RFP12*	2.00^a^	2.08^a^	1.79^a^	0.88^b^	1.03^b^	1.15^b^	0.184	<0.01	0.52	0.18	0.25
*unidentified_[Mogibacteriaceae]*	1.54	1.51	1.44	1.85	1.78	1.84	0.161	0.02	0.92	0.74	0.95
*unidentified_S24-7*	1.33	1.47	1.05	1.25	1.18	0.91	0.223	0.64	0.11	0.07	0.93
*unclassified_Clostridiales*	1.12^bc^	1.14^bc^	1.00^c^	1.48^a^	1.41^a^	1.54^a^	0.098	<0.01	0.51	0.19	0.46
*Butyrivibrio*	1.10^ab^	1.14^a^	0.86^bc^	0.96^ac^	0.98^ac^	0.76^c^	0.087	0.08	0.07	0.01	0.98
*Others*	18.01	18.68	20.33	19.21	19.11	18.98	0.713	0.82	0.27	0.73	0.21

1) CON, control was supplemented without ACB or ICB; ACB was supplemented with 2.4×10^7^ CFU/bottle of ACB; ICB was supplemented with 24 mg/bottle of ICB.

2) pH = pH 5.8 vs. pH 6.5, comparison among treatments of control and CB products at media pH 5.8 and pH 6.5; CB = ACB vs. ICB, comparison among ACB and ICB products at low (pH 5.8) or high media (pH 6.5); Low, comparison among ACB and ICB products at low media pH (5.8); High, comparison among ACB and ICB products at high media pH (6.5); pH×CB, the interaction between media pH and CB products.

a–c Means within a row with different superscripts differ (p<0.05).

CB, *Clostridium butyricum*; CON, control; ACB, active *Clostridium butyricum*; ICB, inactive (heat-killed) *Clostridium butyricum*; SEM, standard error of the mean.
